# Loss of STAT3 in acute myeloid leukemia favors tissue infiltration linked to CXCR4 signaling

**DOI:** 10.1016/j.bneo.2025.100158

**Published:** 2025-08-04

**Authors:** Bernhard Zdársky, Sophie Edtmayer, Agnieszka Witalisz-Siepracka, Stefanie Weiss, Stefanie Boigenzahn, Kerstin Heindl, Safia Zahma, Balázs Győrffy, Richard Moriggl, Dagmar Stoiber

**Affiliations:** 1 Division of Pharmacology, Department of Pharmacology, Physiology and Microbiology, Karl Landsteiner University of Health Sciences, Krems an der Donau, Austria; 2 Institute of Animal Breeding and Genetics, University of Veterinary Medicine Vienna, Vienna, Austria; 3 Department of Bioinformatics, Semmelweis University, Budapest, Hungary; 4 Department of Biophysics, Medical School, University of Pecs, Pecs, Hungary; 5 Cancer Biomarker Research Group, Institute of Molecular Life Sciences, HUN-REN Research Centre for Natural Sciences, Budapest, Hungary; 6 Department of Biosciences and Medical Biology, Paris Lodron University of Salzburg, Salzburg, Austria

**Figure:**
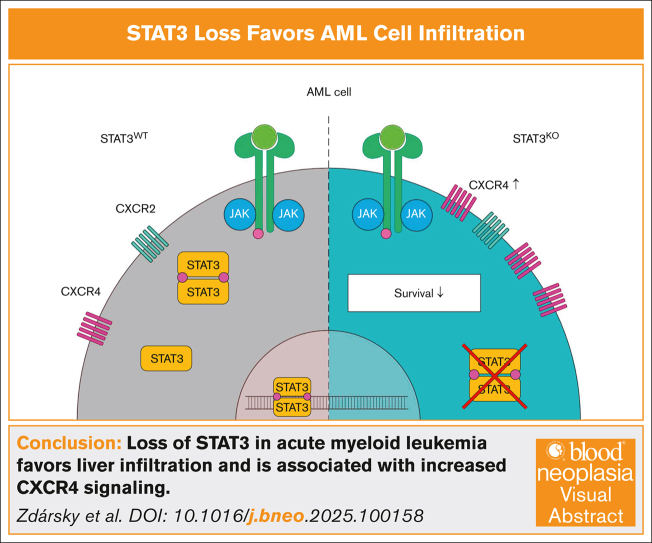

**TO THE EDITOR:**

Acute myeloid leukemia (AML) represents a heterogeneous hematologic malignancy with poor prognosis and high relapse rates.^1^ Recent studies revealed deregulated genes involved in migration, metastasis, or epithelial-mesenchymal transition as key factors in AML, with epithelial-mesenchymal transition signatures serving as independent risk factors.^2^ Signal transducer and activator of transcription 3 (STAT3), is a regulator of fundamental cellular mechanisms, including proliferation, apoptosis, differentiation, metabolism or metastasis.^3^ It has been shown that STAT3 is overactivated in various solid tumors and hematological malignancies. In 44% of patients with AML, STAT3 exhibits constitutive activity, which is associated with short disease-free survival.^4^ However, STAT3 was found to be downregulated in pediatric patients with AML and upregulated in adult patients,^5^ whereas its truncated isoform STAT3β acts as a tumor suppressor.^6^ Furthermore, it was demonstrated that patient-derived AML blasts have variable levels of constitutively tyrosine-phosphorylated STAT3 and that increased cytokine-dependent STAT3 activation is correlated with a better outcome.^7^

To determine the impact of STAT3 in AML, we first explored *STAT3* gene expression differences by reanalyzing the Beat AML^8^ patient cohort data (Figure 1A). Interestingly, patients with mixed-lineage leukemia (MLL)-AF9–driven AML exhibit the lowest *STAT3* levels compared to other subgroups of patients with AML carrying common driver mutations. To gain insight into the impact of STAT3 loss on AML development we established a CRISPR/Cas9–mediated STAT3^KO^ (knockout) in THP-1 and MOLM-13 cells both harboring *t(9;11) (p22;q23)* and expressing the *MLL-AF9* (*KMT2A*-*MLLT3*; *MLLT3-MLL*) fusion gene and HEL cells exhibiting a constitutive STAT3 phosphorylation (*JAK2*^*V617F*^ mutation) but no *MLL-AF9* fusion (Figure 1B; supplemental Figure 1A). Surprisingly, only NSG (*NOD.Cg-Prkdc*^*scid*^*Il2rg*^*tm1Wjl*^*/SzJ)* mice intravenously transplanted with MLL-AF9–positive STAT3^KO^ cells exhibited significantly shorter overall survival (OS) compared to the corresponding STAT3^WT^ (wildtype, nontargeting single guide RNA [sgRNA control]) group. In contrast, STAT3^KO^ in HEL cells did not affect the survival and was comparable to mice injected with the respective control cell line (Figure 1C). Furthermore, mice receiving STAT3^WT^ THP-1 cells displayed higher spleen and body weight compared to animals transplanted with STAT3^KO^ THP-1 cells (supplemental Figure 1B) indicating extramedullary hematopoiesis. In contrast, at the disease end point, the spleen weight of the STAT3^KO^ MOLM-13 group was increased compared to its corresponding control (supplemental Figure 1C), whereas animals transplanted with STAT3^WT^ or STAT3^KO^ HEL cells (supplemental Figure 1D) did not show any significant difference. To analyze the accumulation of myeloid tumor cells in distant organs, cells were isolated from bone marrow (Figure 1D) and spleen (supplemental Figure 1E) and analyzed by flow cytometry. The bone marrow showed similar frequencies of hCD45^+^ THP-1 or HEL cells, but significantly more hCD45^+^ cells in mice transplanted with STAT3^KO^ MOLM-13 cells (Figure 1D). These data indicate that loss of STAT3 accelerates disease development especially in MLL-AF9–driven AML.

**Figure 1.**
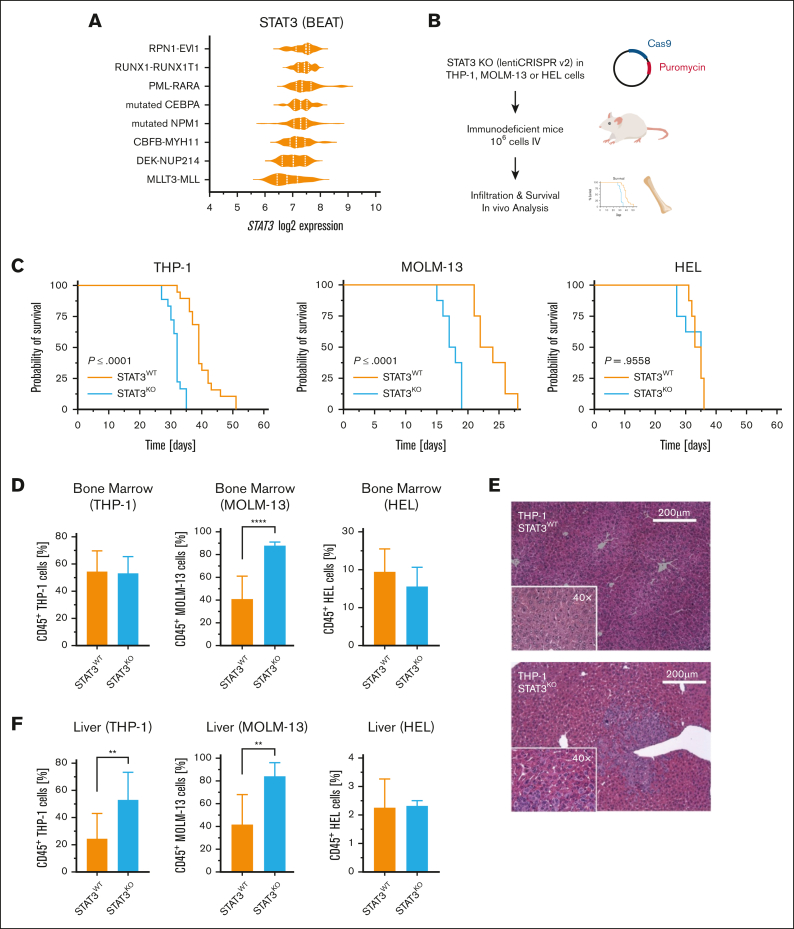
**Loss of STAT3 leads to an accelerated AML progression and aggressive liver infiltration.** (A) Analysis of the Beat AML cohorts shows *STAT3* gene expression in patients harboring common AML-driver mutations (n = 286). (B) AML cells were lentivirally transduced using the lentiCRISPR v2 plasmid, encoding a puromycin resistance gene, the Cas9 endonuclease, and sgRNA targeting STAT3 or a nontargeting control sgRNA. Confirmed single-cell clones were intravenously transplanted into immunocompromised NSG mice. Transplanted recipient mice have been euthanized as soon as they exhibited clinical signs of leukemia and organs were processed for further analysis. (C) Kaplan-Meier plot of mice receiving STAT3^KO^ THP-1 (n = 18), MOLM-13 (n = 8) or HEL (n = 8) cells compared to mice transplanted with wildtype STAT3 cells (n = 18). (D) Percentage of hCD45^+^ AML cells isolated from the bone marrow (THP-1, n = 18; MOLM-13, n = 8; HEL, n = 8). (E) Representative hematoxylin and eosin-stained liver sections of diseased mice receiving THP-1 cells at the time of euthanasia. (F) hCD45^+^ cells isolated from the liver of diseased animals (THP-1, n = 18; MOLM-13, n = 8; HEL, n = 8). Log-rank (Mantel-Cox) test was performed to analyze the survival difference between the 2 groups. Further statistical analysis was performed using Student *t* test. *P* values <.05 were considered statistically significant. ∗*P* < .05, ∗∗*P* < .01, and ∗∗∗*P* < .001. Error bars represent mean ± standard deviation. KO, knockout; WT, wildtype.

Studies in mouse embryonic fibroblasts revealed that *Stat3* loss resulted in a reduced directional cell movement and an abnormal mode of mesoderm migration.^9^ A recent study further underlined that impaired STAT3 signaling is pivotal for cancer metastasis and infiltration, as mice with *PTEN* and *STAT3* deletions rapidly succumbed to metastatic disease.^10^ Surprisingly, and in stark contrast to the control, mice transplanted with STAT3^KO^ THP-1 cells showed a severe liver phenotype (Figure 1E; supplemental Figure 2A-B). Livers of mice transplanted with STAT3^KO^ HEL cells did not display any significant hepatic damage/alterations (supplemental Figure 2A-B). Moreover, we detected significantly more hCD45^+^ THP-1 cells in the liver (Figure 1F). Deletion of STAT3 in HEL cells, however, did not impact cell infiltration into the liver. Loss of STAT3 in MOLM-13 cells resulted in a significant increase of hCD45^+^ cells in the liver (Figure 1F), suggesting that STAT3 influences leukemia progression and organ infiltration in this context.

Chemokines regulate cancer functions, including proliferation and invasion, thereby promoting cancer progression in the tumor microenvironment. Therefore, we analyzed gene expression of 4 chemokine receptors, C-C chemokine receptor type 2 (*CCR2*), *CCR6*, C-X-C motif chemokine receptor 2 (*CXCR2*) and *CXCR4*, linked to liver infiltration and liver disease.^11^ Indeed, all 4 chemokine receptors were significantly upregulated in ex vivo sorted STAT3^KO^ THP-1 cells that had been passaged in mice and were isolated from diseased animals (supplemental Figure 2C). Due to the described role of CXCR4 and CXCR2 in mediating the interaction between AML cells and their microenvironment,^12^ we further analyzed their protein expression pattern. CXCR2 was present in low abundance and protein expression remained unchanged in all AML cell lines after loss of STAT3 (supplemental Figure 2D). Analysis of cytoplasmic CXCR4 in in vitro cultured leukemia cells revealed no significant differences across cell lines (supplemental Figure 2E). However, ex vivo analyzed STAT3^KO^ THP-1 and MOLM-13 cells expressed significantly more surface CXCR4, whereas barely any expression was detected in STAT3^KO^ HEL cells (Figure 2A-B).

**Figure 2.**
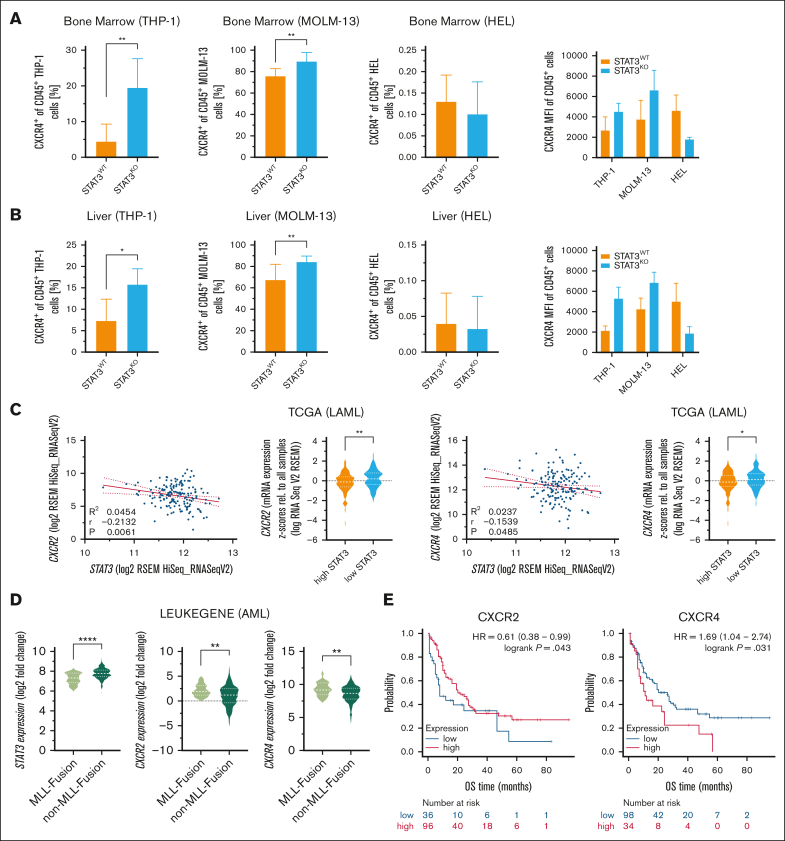
**STAT3^KO^ leads to increased CXCR4 expression which correlates with worse OS and negatively with *STAT3* expression in patients with AML.** Flow cytometry quantification of CXCR4^+^ of hCD45^+^ ex vivo THP-1, MOLM-13 and HEL cells from the (A) bone marrow and the (B) liver (THP-1, n = 18; MOLM-13, n = 8; HEL, n = 8). (C) Gene expression correlation of *STAT3* and *CXCR2/CXCR4* in patients with AML from the TCGA data set (n = 190). Red line represents linear regression with 95% confidence interval. (D) *STAT3*, *CXCR2*, and *CXCR4* gene expression analysis of patients with AML of the LEUKEGENE AML study with and without MLL fusion genes (n = 365). (E) Kaplan-Meier plots showing OS of patients with AML expressing low and high *CXCR2* or *CXCR4* (n = 132). Patients were stratified according to the best cutoff value (kmplot.com). Statistical analysis was performed using Student *t* test. *P* values <.05 were considered statistically significant. ∗*P* < .05, ∗∗*P* < .01, and ∗∗∗*P* < .001. Error bars represent mean ± standard deviation. HR, hazard ratio; KO, knockout; TCGA, The Cancer Genome Atlas; WT, wildtype.

In a feed-forward loop, *CXCR2* expression can be directly regulated by STAT3, and vice versa, CXCR2 can activate STAT3 signaling^13^ and a CXCR4-STAT3 axis was discussed in various tissues and cancers including chronic lymphocytic leukemia.^14^, ^15^, ^16^, ^17^ The connection of STAT3-dependent regulation of CXCR2 and CXCR4 has not been described so far in AML. Because STAT3 loss upregulates the expression of chemokine receptors, we analyzed publicly accessible patient data from The Cancer Genome Atlas,^18^ LEUKEGENE AML,^19^ St. Jude^20^ cohorts, and Beat AML,^8^ to assess clinical relevance. Importantly, analysis of the The Cancer Genome Atlas gene expression data set revealed that *STAT3* expression negatively correlates with *CXCR2* and *CXCR4* but not with *CCR2* or *CCR6* expression in patients with AML (Figure 2C; supplemental Figure 3A). In agreement with our data, re-analysis of human *MLL*-rearranged AML patient data from the LEUKEGENE AML study showed higher *STAT3* and lower *CXCR2* and *CXCR4* expression in AML blasts of patients carrying *MLL*-rearrangements compared to patients without *MLL*-rearrangements (Figure 2D). Additional analyses of gene expression in the St. Jude (supplemental Figure 3B) and Beat AML (supplemental Figure 3C) cohorts provided further context for our findings, with results showing some overlap but also variability. To gain more patient relevance, we used an additional RNA-sequencing data set and correlated gene expression to the OS of 132 patients with AML. Although higher *CCR2* expression correlated with poorer OS, *CCR6* expression did not impact OS in this patient cohort (supplemental Figure 3D). Remarkably and in line with our data, *CXCR4* but not *CXCR2* correlated with poorer OS in patients with AML (Figure 2E). However, decreased STAT3 levels could also lead to dysregulation of other pathways that enhance CXCR4 expression. In conclusion, we identified STAT3 as a relevant modulator in invasive infiltration in AML, potentially associated with MLL-rearrangements. Although our results highlight the potential role of interleukin-6/STAT3 signaling in AML, further validation is needed before considering STAT3 and CXCR4 as prognostic markers for treatment management. Our findings suggest careful consideration before the use of interleukin-6/STAT3 signaling inhibitors in AML therapy.

Animal experiments were approved by the animal ethics committee of the Medical University of Vienna and the Austrian Ministry of Education, Science and Research and performed according to the Federation of European Laboratory Animal Science Associations (FELASA) guidelines.

**Conflict-of-interest disclosure:** The authors declare no competing financial interests.

## Supplementary Material

Supplemental Methods, Tables, Figures, and References
