# Supplementary material for: Loss of STAT3 in acute myeloid leukemia favors tissue infiltration linked to CXCR4 signaling
Source: Blood Neoplasia. 2025 Aug 4;2(4):100158. doi: 10.1016/j.bneo.2025.100158 (PMC12515738; doi:10.1016/j.bneo.2025.100158)
Supplement: Supplemental Methods, Tables, Figures, and References [file BNEO_NEO-2024-000538-mmc1.pdf]

Supplementary Data

Loss of STAT3 in Acute Myeloid Leukemia Favors Tissue Infiltration Linked to CXCR4 Signaling

Material and Methods

AML patients

For gene expression analysis, the publicly available gene expression data from AML patients included in The Cancer Genome Atlas (TCGA),[1] BEAT AML,[2] St. Jude[3] and LEUKEGENE (GSE62190, GSE66917, GSE67039) was used.[4] To explore the impact of gene expression on relapse-free survival rates among the AML patients, we conducted a Cox regression analysis. To ensure comprehensive analysis without overlooking significant details due to the selection of a specific gene expression threshold, we evaluated all potential values ranging from the lowest to the highest quartiles of expression levels for the combined signature. However, the extensive number of tests performed may yield false positive results. To address this issue, we calculated the False Discovery Rate (FDR) to adjust for multiple hypothesis testing. To visually depict the variations in survival rates associated with gene expression, we generated Kaplan-Meier survival plots. These plots utilize the threshold values identified in the univariate analysis to illustrate the impact of gene expression levels on patient survival outcomes.

Animal studies

Animal experiments were approved by the Animal Ethics Committee of the Medical University of Vienna and the Austrian Ministry of Education, Science and Research and performed according to the FELASA guidelines. Female age-matched (8–12 weeks) animals were bred and kept under pathogen-free conditions at the Institute of Pharmacology, Medical University of Vienna (Vienna, Austria).

Cell lines and cell culture

Unless otherwise stated, all cell culture reagents were purchased from Gibco (Thermo Fisher Scientific, Waltham, MA, USA). THP-1, HEL, MOLM-13 cells were purchased from the American Type Culture Collection (ATCC, Manassas, VA, USA). AML cells were routinely maintained in Roswell Park Memorial Institute (RPMI) 1640 medium containing 10% fetal bovine serum (FBS), phenol red, 100 U/mL penicillin, 0.1 mg/mL streptomycin, and 2 mM glutamine. All cells were cultured at 37°C with 5% CO_2. All cell lines were regularly tested for mycoplasma contamination using the MycoAlert Detection Kit (Lonza, Verviers, Belgium). Cells

were treated with ruxolitinib (LC Laboratories, MA, USA) or cytarabine (Seleckchem, Houston, TX, USA) as indicated.

CRISPR/Cas9 knockout cell lines

Single guide RNA (sgRNA) sequences for CRISPR/Cas9 were designed using CHOPCHOP (version 3) (32) and cloned into lentiCRISPR v2 (Addgene #52961) (33). The following sgRNA oligonucleotides were used: NT-sgRNA: 5'-CACCGACGGAGGCTAAGCGTCGCAA-'3; STAT3-sgRNA1: 5'-CACCGCATTCGACTCTTGCAGGAAG-'3; STAT3-sgRNA2: 5'-CACCGCAGCTTGACACACGGTACC-'3. All sequences were verified by sequencing prior to experimental use. For lentiviral transduction, Lenti-X 293T cells (Takara Bio, Kusatsu, Japan) were transfected with lentiCRISPR v2 containing respective sgRNAs together with psPAX2 (Addgene #12260) and pMD2.G (Addgene #12259) using calcium phosphate co-precipitation. Cells were spin-oculated (1000 g for 90 min) with retroviral supernatant supplemented with polybrene (Sigma-Aldrich, St. Louis, MO, USA). Transduced cells were treated with 2-4 µg/ml puromycin (Sigma-Aldrich), and single cell-derived clones were acquired via serial dilution. Successful genome edits were analyzed by Western blot. Unless otherwise stated, all experiments were carried out with sgRNA1.

Flow cytometry

Single-cell suspensions were incubated with antibodies 1:100 - 1:200 in phosphate-buffered saline (PBS) containing 2% FBS and Fc-receptor blocker (BioLegend, San Diego, CA, USA) for 30 min on ice then washed. For *ex vivo* experiments, $hCD45^+$ AML cells were sorted using a BD FACSymphonyTM S6 Cell Sorter (BD Bioscience San Jose, CA, USA). Cytoplasmic CXCR4 expression was analyzed using two specific CXCR4 antibodies. Prior to staining, cells were fixed and permeabilized with the eBioscience™ FOXP3/Transcription Factor Staining Kit (#00-5523-00) following the manufacturer's instructions which allows intracellular staining. Cytoplasmic CXCR4 levels were calculated by subtracting the surface CXCR4 expression from the total CXCR4 expression, providing a quantitative measure of intracellular CXCR4 levels. Antibodies are listed in Supplementary Table 2.

Quantitative PCR (qPCR)

RNA was isolated from cells using QIAzol Lysis Reagent (Qiagen, Hilden, Germany) or for patient samples using the RNeasy Mini Kit (Qiagen) and reverse transcription-PCR was performed using RevertAid H Minus First Strand cDNA Synthesis Kit (Thermo Fisher Scientific) according to the manufacturer's instructions. qPCR was performed in duplicates using SsoAdvancedTM Universal SYBR® Green Supermix (Bio-Rad, Hercules, CA, USA) and

measured on a qTower3 (Analytik Jena, Jena, Germany). *GAPDH* was used as a housekeeping gene to calculate relative gene expression. Primers are listed in Supplementary Table 1**.**

Western blot analysis

Cells were lysed in Laemmli Buffer (Bio-Rad), and samples were denaturated for 10 min at 95°C and equal protein amounts were separated by SDS-PAGE using 10% polyacrylamide. Proteins were analyzed after blotting onto 0.45 µm PVDF membranes (Carl Roth), which were subsequently blocked with 5% skim milk powder in PBS containing 0.1% Tween20 (Carl Roth). Primary antibodies were incubated overnight at 4°C with the PVDF membranes, followed by incubation with secondary antibody for 1 h at room temperature. Proteins were detected using SuperSignalTM West Femto Maximum Sensitivity Substrate (Thermo Fisher Scientific) visualized by a ChemiDoc Imager (Bio-Rad). Antibodies are listed in Supplementary Table 2.

Xenograft models in NSG mice and organ processing

1×10^6 cells were transplanted into immunocompromised NOD.Cg-PrkdcscidIl2rgtm1Wjl/SzJ (The Jackson Laboratory, Bar Harbor, ME) mice via the tail vein. Disease progression was monitored over time and survival was evaluated via Kaplan-Meier analysis. The transplanted recipient mice were euthanized at the first signs of disease. The spleens were isolated and mashed through a 70 µm cell strainer, followed by red blood cell lysis (Lonza). Cells infiltrated in the bone marrow were harvested from hind legs and cells infiltrated into the liver were separated from hepatocytes using a Percoll (Cytivia US, Massachusetts, Marlborough) gradient.

Statistical analysis

Data were analyzed using GraphPad Prism 10 software. Kaplan–Meier and log-rank (Mantel-Cox) statistical evaluation was used to analyze survival. For comparing two groups unpaired Students t-test was employed. Outliers were excluded based on Grubbs' test (significance level alpha = 0.05). Error bars represent mean ± standard deviations (SD). p-values are indicated as p ≤0.05:*, ≤ 0.01:**, ≤ 0.001:*** and ≤ 0.0001:****. The absence of a p-value or asterisk indicates the lack of statistical significance.

Supplementary Tables

Supplementary Table 1: qPCR Primer set

Gene	Primer sequence (5' → 3')
CCR2 FW	CAGGTGACAGAGACTCTTGGGA
CCR2 RV	GGCAATCCTACAGCCAAGAGCT
CCR6 FW	CTCTGGCTTTTGTTCACTGCTGC
CCR6 RV	AGCCCACAGTATTGGCAGAGCA
CXCR2 FW	TCCGTCACTGATGTCTACCTGC
CXCR2 RV	TCCTTCAGGAGTGAGACCACCT
CXCR4 FW	CTCCTCTTTGTCATCACGCTTCC
CXCR4 RV	GGATGAGGACACTGCTGTAGAG
GAPDH FW	TCTCCTCTGACTTCAACAGCG
GAPDH RV	ACCACCCTGTTGCTGTAGCC

Supplementary Table 2: Antibodies

Target	Company	Article# / clone	Method
STAT3	Cell Signaling	12640	Western blot
STAT1	Cell Signaling	9172	Western blot
STAT5	Cell Signaling	94205	Western blot
β-ACTIN	Cell Signaling	4967	Western blot
anti-rabbit IgG HRP-linked	Cell Signaling	7074P2	Western blot
hCD45	BioLegend	304021/HI30	Flow cytometry
CD184 (CXCR4)	eBioscience	12-9999-41/12G5	Flow cytometry
CD184 (CXCR4)	BioLegend	306509/12G5	Flow cytometry
CD182 (CXCR2)	BioLegend	320723/5E8	Flow cytometry

Supplementary Figures

Figure S1: Loss of STAT3 leads to an accelerated AML progression

(A) Representative Western blot showing complete CRISPR/Cas9-mediated *STAT3* knockout ($STAT3^{KO}$) and quantification of the protein expression levels of STAT3, STAT1, and STAT5 relative to the non-targeting sgRNA control (NT), normalized to β-ACTIN (n=3). Body, spleen and spleen-to-body weight ratio of diseased animals at the timepoint of euthanasia previously transplanted with WT or STAT3-deficient (**B**) THP-1 (n=18), (**C**) MOLM-13 (n=8) or (**D**) HEL (n=8) cells. (**E**) $hCD45^+$ AML cells isolated from the spleen (THP-1, n=18; MOLM-13, n=8; HEL, n=8). Student's *t-test*. p values < 0.05 were considered statistically significant. *p < 0.05, **p < 0.01, ***p < 0.001. Error bars represent mean ± standard deviation. KO: knockout; STAT3: signal transducer and activator of transcription 3; WT: wildtype.

Figure S2: STAT3^{KO} leads to increased CXCR4 expression and aggressive liver infiltration

(**A**) Representative pictures of livers isolated from diseased mice, transplanted with STAT3^{KO} THP-1, MOLM-13 or HEL cells at the time point of euthanasia. (**B**) Liver weight of diseased animals at euthanasia (THP-1 n=18, HEL n=8, MOLM-13 n=8). (**C**) qPCR analysis of *ex vivo* WT and STAT3^{KO} THP-1 cells (n=4). (**D**) Flow cytometry quantification of CXCR2^{+} *in vitro* cultured THP-1, HEL and MOLM-13 cells. (**E**) Flow cytometry quantification of cytoplasmic CXCR4. Statistical analysis was performed using Student's *t-test*. p values < 0.05 were considered statistically significant. *p < 0.05, **p < 0.01, ***p < 0.001. Error bars represent mean ± standard deviation. CCR: C-C chemokine receptor; CXCR: C-X-C motif chemokine receptor; KO: knockout; STAT3: signal transducer and activator of transcription 3; WT: wildtype.

Figure S3: *STAT3* expression does not correlate with *CCR2* and *CCR6* expression in AML patients

(**A**) Gene expression correlation of *STAT3* and *CCR2 or CCR6* in AML patients from TCGA dataset (n=190). The red line represents the linear regression with a 95% confidence interval. (**B**) Analysis of the BEAT AML cohort shows *CXCR2 and CXCR4* gene expression in common AML driver mutations (n=286). (**C**) Analysis of the St. Jude AML cohort shows STAT3, *CXCR2 and CXCR4* gene expression in patients with *KMT2A* mutations (n=69). Gene expression correlation of *STAT3* (**D**) Kaplan-Meier plots showing overall survival of AML patients expressing low and high *CCR2, CCR6,* (n=132). Patients were stratified according to the best cut-off value (kmplot.com). AML: acute myeloid leukemia; CCR: C-C Chemokine receptor; OS: overall survival; STAT3: signal transducer and activator of transcription 3; TCGA: The Cancer Genome Atlas.

References

1. Cancer Genome Atlas Research N, Ley TJ, Miller C, et al. Genomic and epigenomic landscapes of adult de novo acute myeloid leukemia. *N Engl J Med*. May 30 2013;368(22):2059-74. doi:10.1056/NEJMoa1301689

2. Tyner JW, Tognon CE, Bottomly D, et al. Functional genomic landscape of acute myeloid leukaemia. *Nature*. Oct 2018;562(7728):526-531. doi:10.1038/s41586-018-0623-z

3. Umeda M, Ma J, Huang BJ, et al. Integrated Genomic Analysis Identifies UBTF Tandem Duplications as a Recurrent Lesion in Pediatric Acute Myeloid Leukemia. *Blood Cancer Discov*. May 5 2022;3(3):194-207. doi:10.1158/2643-3230.BCD-21-0160

4. Lavallee VP, Baccelli I, Krosl J, et al. The transcriptomic landscape and directed chemical interrogation of MLL-rearranged acute myeloid leukemias. *Nat Genet*. Sep 2015;47(9):1030-7. doi:10.1038/ng.3371

Figure S1

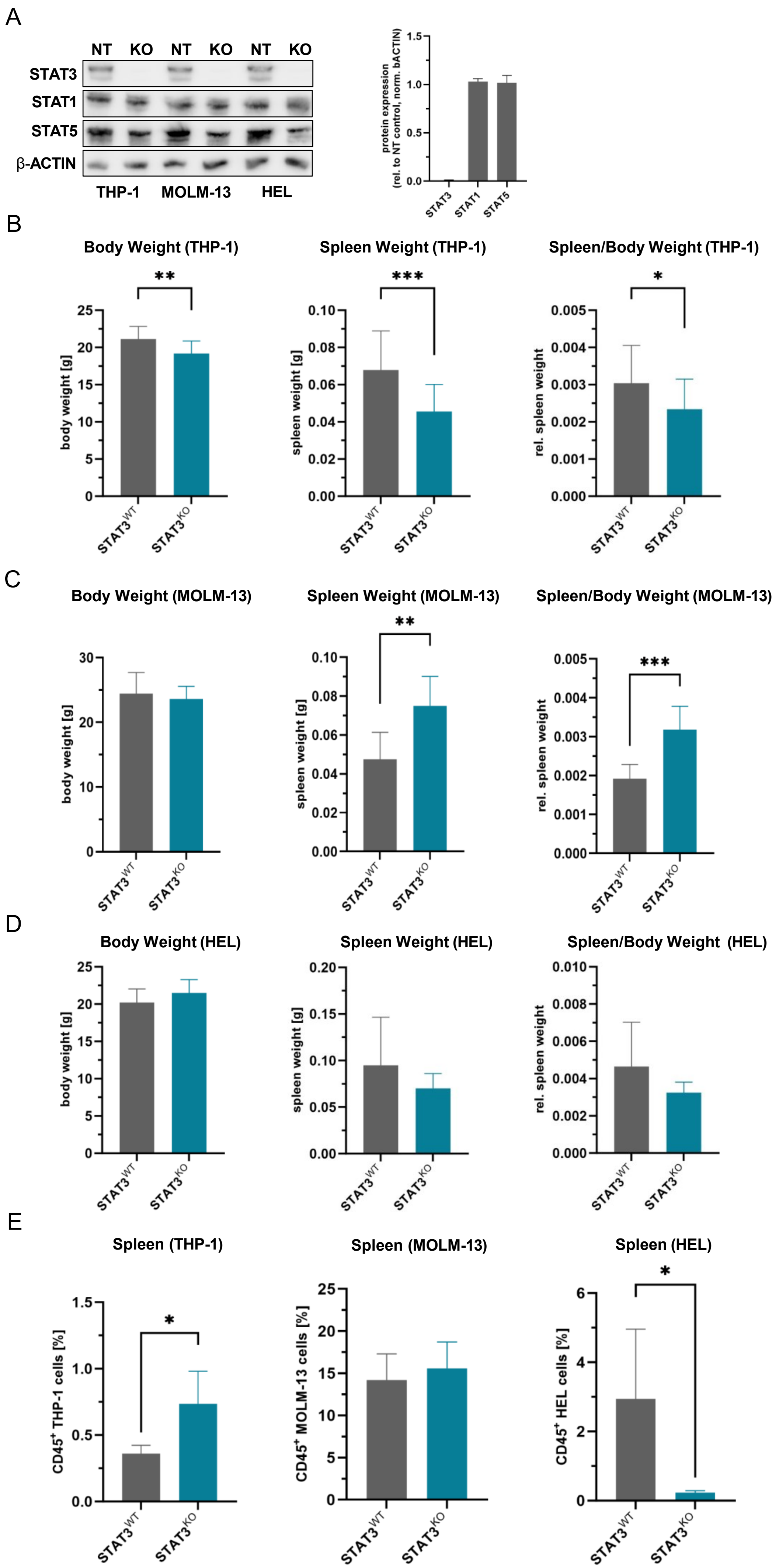
A
NT KO NT KO NT KO
STAT3
STAT1
STAT5
β-ACTIN
THP-1 MOLM-13 HEL
protein expression (rel. to NT control, norm. bACTIN)
STAT3 STAT1 STAT5
B
Body Weight (THP-1)
Spleen Weight (THP-1)
Spleen/Body Weight (THP-1)
body weight [g]
spleen weight [g]
rel. spleen weight
STAT3WT STAT3KO
C
Body Weight (MOLM-13)
Spleen Weight (MOLM-13)
Spleen/Body Weight (MOLM-13)
D
Body Weight (HEL)
Spleen Weight (HEL)
Spleen/Body Weight (HEL)
E
Spleen (THP-1)
Spleen (MOLM-13)
Spleen (HEL)
CD45+ THP-1 cells [%]
CD45+ MOLM-13 cells [%]
CD45+ HEL cells [%]

Figure S2

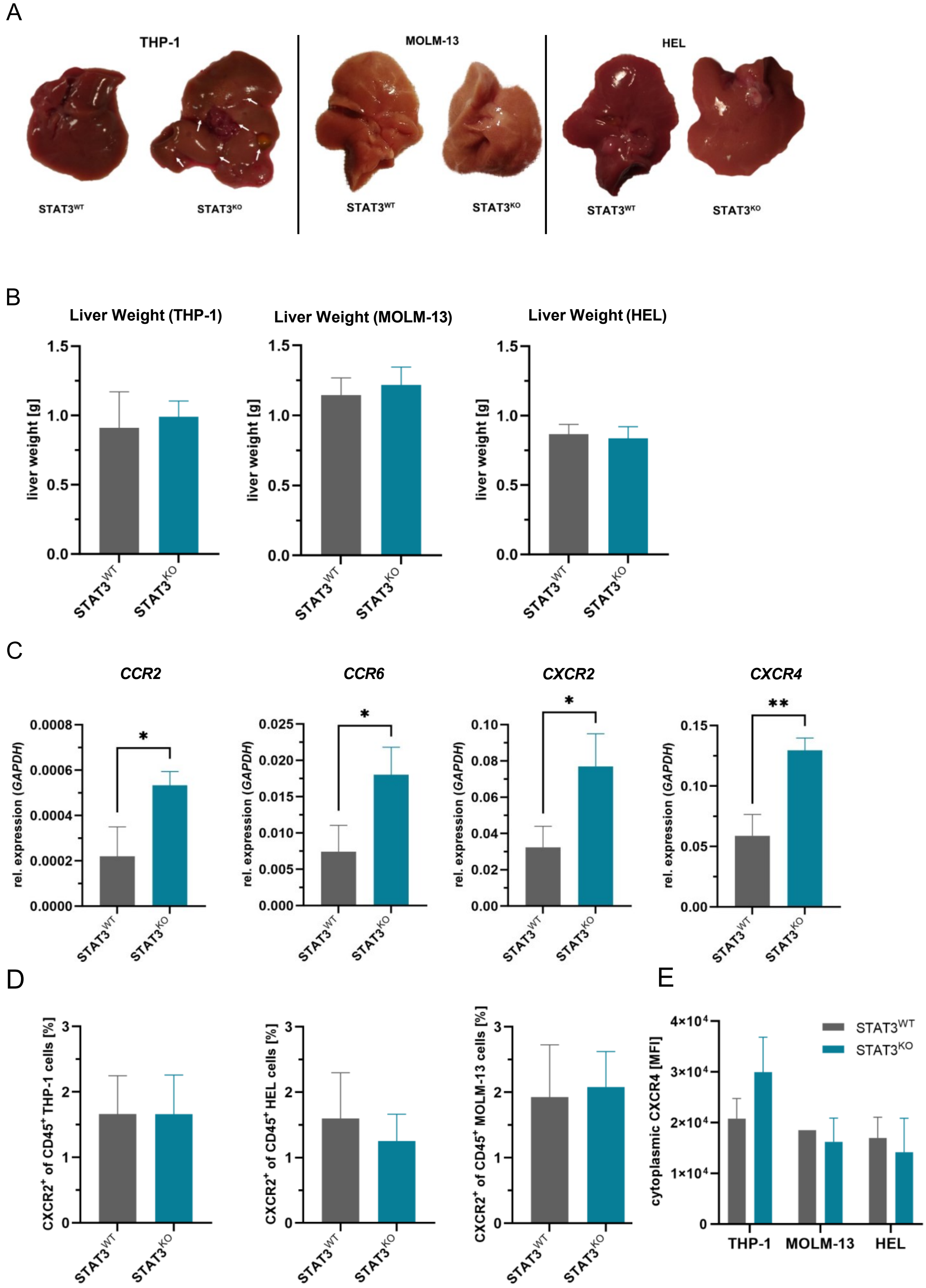
A
THP-1
MOLM-13
HEL
STAT3WT
STAT3KO
B
Liver Weight (THP-1)
Liver Weight (MOLM-13)
Liver Weight (HEL)
liver weight [g]
C
CCR2
CCR6
CXCR2
CXCR4
rel. expression (GAPDH)
D
CXCR2+ of CD45+ THP-1 cells [%]
CXCR2+ of CD45+ HEL cells [%]
CXCR2+ of CD45+ MOLM-13 cells [%]
E
cytoplasmic CXCR4 [MFI]
STAT3WT
STAT3KO
THP-1
MOLM-13
HEL

Figure S3

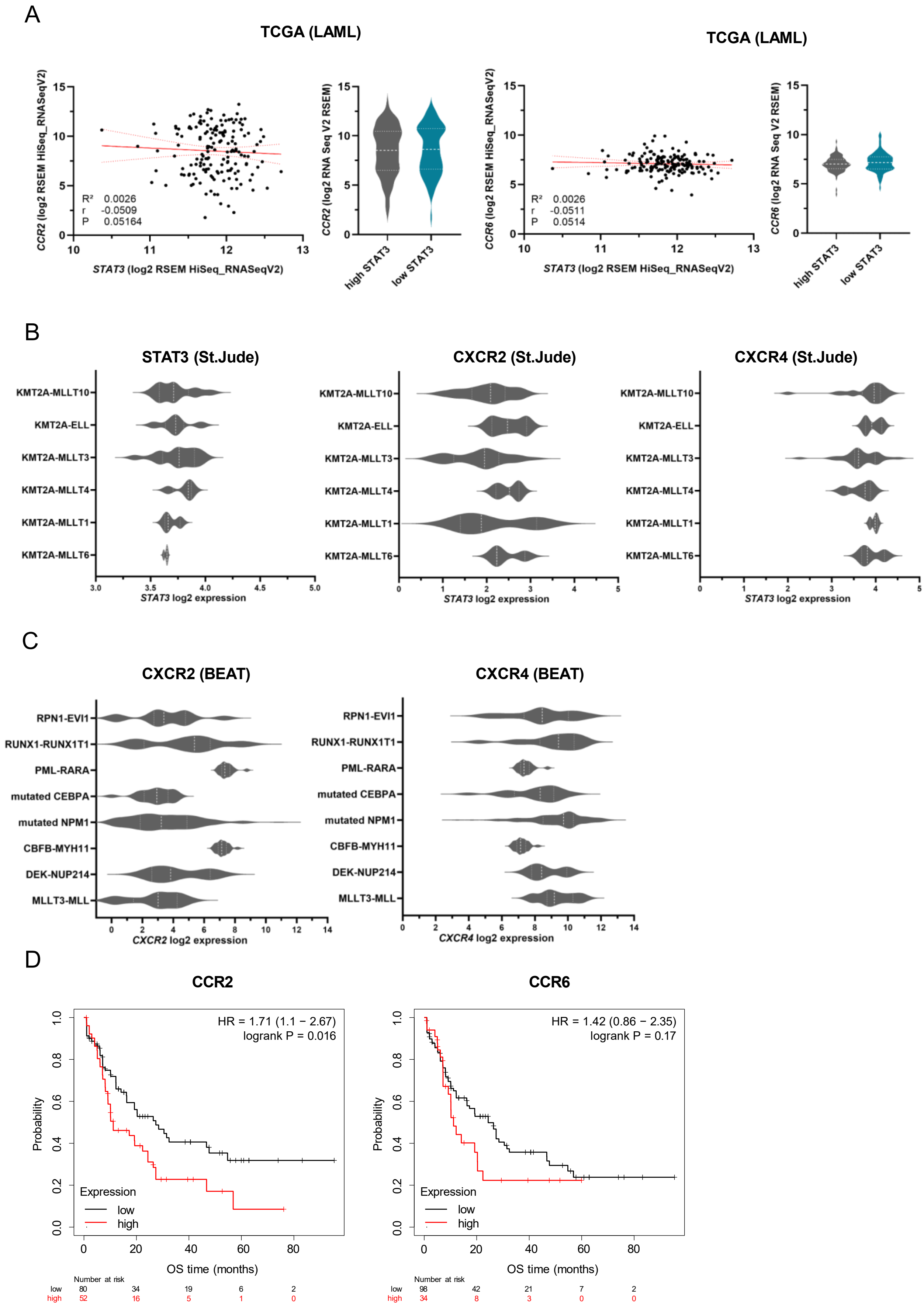

A
TCGA (LAML)
CCR2 (log2 RSEM HiSeq_RNASeqV2)
STAT3 (log2 RSEM HiSeq_RNASeqV2)
R² 0.0026
r -0.0509
P 0.05164
CCR2 (log2 RNA Seq V2 RSEM)
high STAT3
low STAT3
TCGA (LAML)
CCR6 (log2 RSEM HiSeq_RNASeqV2)
STAT3 (log2 RSEM HiSeq_RNASeqV2)
R² 0.0026
r -0.0511
P 0.0514
CCR6 (log2 RNA Seq V2 RSEM)
high STAT3
low STAT3
B
STAT3 (St.Jude)
CXCR2 (St.Jude)
CXCR4 (St.Jude)
KMT2A-MLLT10
KMT2A-ELL
KMT2A-MLLT3
KMT2A-MLLT4
KMT2A-MLLT1
KMT2A-MLLT6
STAT3 log2 expression
C
CXCR2 (BEAT)
CXCR4 (BEAT)
RPN1-EVI1
RUNX1-RUNX1T1
PML-RARA
mutated CEBPA
mutated NPM1
CBFB-MYH11
DEK-NUP214
MLLT3-MLL
CXCR2 log2 expression
CXCR4 log2 expression
D
CCR2
CCR6
HR = 1.71 (1.1 − 2.67)
logrank P = 0.016
HR = 1.42 (0.86 − 2.35)
logrank P = 0.17
Probability
Expression
low
high
OS time (months)
Number at risk
low 80 34 19 6 2
high 52 16 5 1 0
low 98 42 21 7 2
high 34 8 3 0 0
